# Efficacy and Safety of Ultrasound-Guided Transmuscular Quadratus Lumborum Block as an Adjunct to Local Infiltration Analgesia After Primary Total Hip Arthroplasty: A Retrospective Before-After Comparative Study

**DOI:** 10.7759/cureus.108057

**Published:** 2026-04-30

**Authors:** Kazuki Hirose, Tomonori Tetsunaga, Kazuki Yamada, Tomohiro Inoue, Tomoko Tetsunaga, Toshifumi Ozaki

**Affiliations:** 1 Department of Orthopaedic Surgery, Kochi Health Sciences Center, Kochi, JPN; 2 Department of Musculoskeletal Health Promotion, Faculty of Medicine, Dentistry and Pharmaceutical Sciences, Okayama University, Okayama, JPN; 3 Department of Medical Materials for Musculoskeletal Reconstruction, Faculty of Medicine, Dentistry and Pharmaceutical Sciences, Okayama University, Okayama, JPN; 4 Department of Orthopaedic Surgery, Okayama UIniversity Hospital, Okayama, JPN; 5 Department of Orthopaedic Surgery, Graduate School of Medicine, Dentistry and Pharmaceutical Sciences, Okayama University, Okayama, JPN

**Keywords:** intravenous patient-controlled analgesia, local infiltration analgesia, postoperative nausea and vomiting, postoperative pain, qlb3, quadratus lumborum block, regional anesthesia, total hip arthroplasty

## Abstract

Background: Total hip arthroplasty (THA) is associated with substantial postoperative pain, which can delay early mobilization and recovery. Quadratus lumborum block (QLB), originally developed for abdominal surgery, has been adapted for hip procedures; however, the clinical benefit and safety of the transmuscular approach (QLB3) remain uncertain. We aimed to evaluate the efficacy and safety of QLB3 in patients undergoing THA.

Methods: We conducted a single-center retrospective cohort study of 93 patients (100%) who underwent primary THA at our institution between February 2021 and February 2022. Patients received QLB3 plus local infiltration analgesia (LIA) (QLB3+LIA group, n = 45 (51.6%)) or LIA alone (LIA group, n = 48 (48.4%)). The primary outcome was postoperative pain intensity assessed using the numeric rating scale at 24 and 48 hours. Secondary outcomes included intravenous patient-controlled analgesia (IV-PCA) consumption within 48 hours and the incidence of postoperative nausea and vomiting (PONV) within 48 hours.

Results: Baseline demographic and surgical characteristics were similar between groups. Mean (SD) pain scores were lower in the QLB3+LIA group at 24 hours (2.4 ± 1.6 vs. 4.2 ± 2.1) and 48 hours (2.4 ± 2.0 vs. 4.3 ± 2.1). IV-PCA consumption within 48 hours was lower in the QLB3+LIA group (10.1 ± 13.2 mL) than in the LIA group (19.7 ± 20.8 mL). PONV occurred less frequently in the QLB3+LIA group (6/45 (13.3%)) than in the LIA group (16/48 (33.3%)). No block-related complications, including nerve palsy, were observed.

Conclusions: QLB3 combined with LIA was associated with reduced postoperative pain, lower IV-PCA consumption, and fewer PONV events after THA, without observed neurologic complications. These findings support the use of QLB3 as part of multimodal analgesia and warrant confirmation in prospective randomized trials.

## Introduction

The incidence of total hip arthroplasty (THA) has increased in recent years, alongside considerable advances in surgical techniques and perioperative care [1-3]. Nevertheless, various complications persist, including chronic postsurgical pain (CPSP). CPSP is a multifactorial condition defined as pain that (1) persists for at least three months after surgery; (2) is absent preoperatively or differs in character or intensity from preoperative pain; (3) is confined to the surgical site or adjacent anatomical regions; and (4) cannot be attributed to other causes, such as malignancy recurrence or postoperative infection [4]. CPSP may lead to delayed mobilization and functional recovery, deep vein thrombosis, and prolonged hospitalization [5]. Reported risk factors include preoperative factors such as female sex, younger age, central sensitization, catastrophizing, mental health issues, and multiple pain sites, as well as postoperative factors including inadequate rehabilitation, insufficient pain control, and complications such as infection or bleeding [6,7].

Among these, postoperative rehabilitation and pain management are particularly important. Approaches for controlling acute postoperative pain after THA include intravenous patient-controlled analgesia (IV-PCA), continuous epidural analgesia, local infiltration analgesia (LIA), femoral nerve block, sciatic nerve block, lumbar plexus block, and quadratus lumborum block (QLB) [8-11]. Each method has specific advantages and limitations. For example, IV-PCA and epidural analgesia can cause hypotension, respiratory depression, nausea or vomiting, and urinary retention [12], whereas nerve blocks may carry risks of nerve injury and muscle weakness [13,14]. In contrast, QLB is an interfascial block that theoretically avoids direct nerve injury and is less likely to cause postoperative muscle weakness.

Originally developed for abdominal surgery [15-17], QLB has more recently been applied in hip surgery. However, evidence for its use in the hip region remains limited, and no established consensus exists [18,19]. QLB can be performed via three approaches: QLB1 and QLB2 primarily block the anterior rami from T7 to L1 [17,20], whereas QLB3 extends coverage to T7-L2 [21,22]. Although several studies suggest that QLB can provide prolonged postoperative analgesia [23-25], current data remain insufficient to draw definitive conclusions. Therefore, we conducted a retrospective before-after comparative study to evaluate the efficacy and safety of ultrasound-guided transmuscular QLB3 as an adjunct to LIA after primary THA. The primary outcome was movement-evoked pain intensity assessed by the numeric rating scale (NRS) at 24 and 48 hours postoperatively. Secondary outcomes included cumulative IV-PCA consumption within 48 hours, the incidence of postoperative nausea and vomiting (PONV) within 48 hours, and hip-specific patient-reported outcomes at discharge.

## Materials and methods

Ethical statement

This retrospective cohort study was conducted in accordance with the ethical principles of the Declaration of Helsinki. Ethical approval was obtained from the Ethics Committee of Okayama University Hospital (Approval No.: K2603-051). As only de-identified data collected during routine clinical care were analyzed and no interventions beyond standard practice were performed, the Ethics Committee waived the requirement for written informed consent. All patient data were handled according to institutional data security protocols, and only anonymized datasets were used for statistical analyses.

Study population

We retrospectively assessed clinical outcomes after primary THA according to postoperative analgesic strategy. This study was a retrospective before-after comparative study. In September 2021, our institution introduced ultrasound-guided transmuscular QLB3 as an adjunct to the existing postoperative analgesic protocol for primary THA; therefore, group allocation was determined by the time period (February 2021-August 2021 vs September 2021-February 2022), rather than by randomization. All consecutive patients undergoing primary THA during the study period were screened for eligibility. Patients were excluded if they did not receive LIA as part of the institutional multimodal analgesia protocol or if postoperative outcome assessments were incomplete. Patients were divided into two groups: 48 cases who underwent LIA from February 2021 to August 2021 (LIA group) and 45 cases who underwent QLB plus LIA from September 2021 to February 2022 (QLB3+LIA group). All eligible consecutive patients in each time period were included, which resulted in similar group sizes (48 vs 45). No matching or selective sampling was performed. Perioperative anesthesia, rehabilitation, and analgesic protocols were unchanged during the study period, except for the introduction of QLB3.

Surgery

All procedures were performed by two experienced hip surgeons. Surgical approaches included the posterior approach, direct lateral approach (Hardinge), and anterolateral approach (modified Watson-Jones or anterolateral spine). Cementless implants were used in all cases. Beginning on postoperative day 1, all patients followed a standardized rehabilitation program, including range-of-motion exercises, muscle strengthening, and full weight-bearing ambulation as tolerated.

Quadratus lumborum block

**Figure 1:**
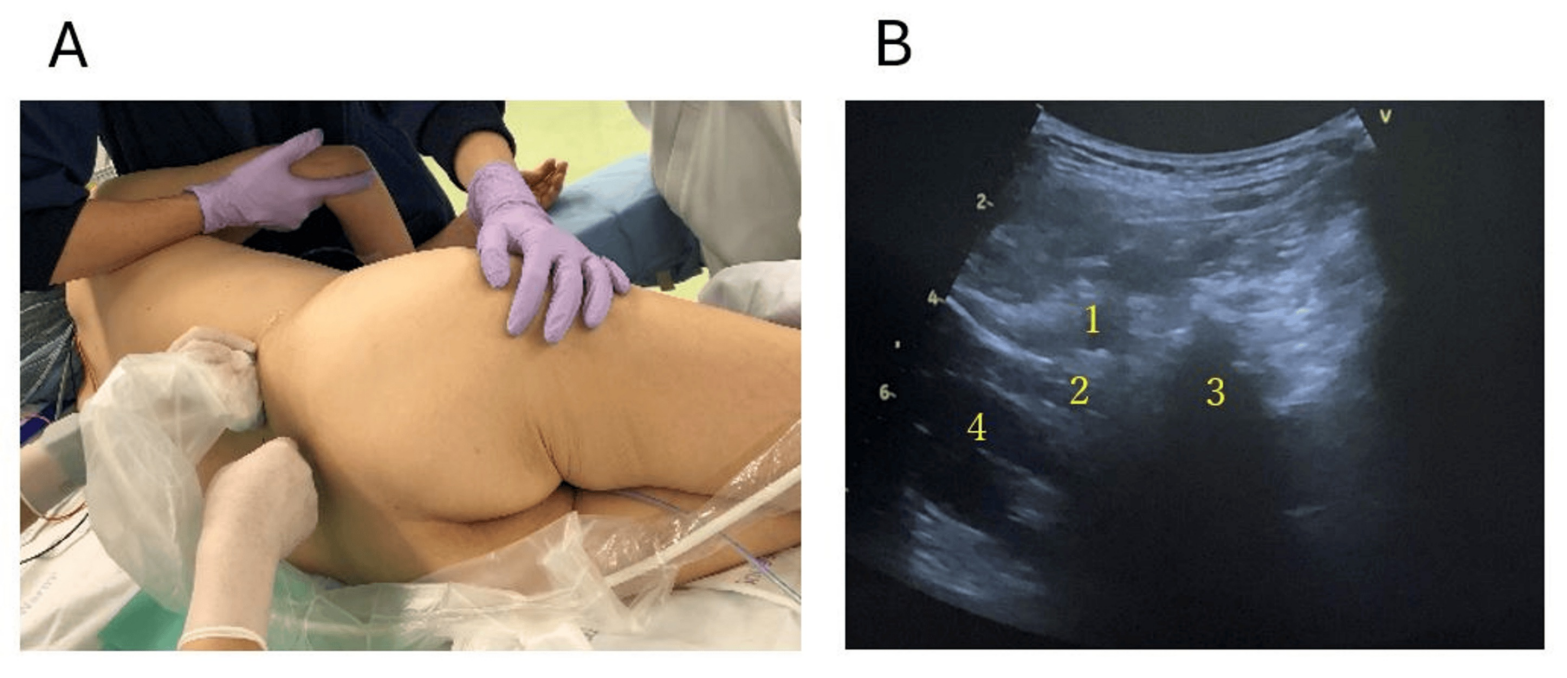
Quadratus lumborum block (A) Photograph of patient positioning and ultrasound transducer placement for QLB3. (B) Ultrasound image of QLB3, showing injection of the anesthetic solution between the quadratus lumborum and psoas major muscles. Labels: (1) quadratus lumborum muscle, (2) psoas major muscle, (3) transverse process, and (4) kidney. QLB: Quadratus lumborum block.

For patients in the QLB3+LIA group, ultrasound-guided QLB3 was performed after induction of general anesthesia with patients in the lateral decubitus position (Figure 1). The procedure was conducted by the attending anesthesiologist under the supervision of an experienced regional anesthesia specialist. The supervising physician was a regional anesthesia specialist with extensive experience in ultrasound-guided truncal blocks, including QLB. Following the standard QLB3 technique, a curved 2-5 MHz ultrasound transducer was placed transversely above the iliac crest at the midaxillary line. On this view, the quadratus lumborum appears anterolateral to the L3-L4 transverse processes, bordered anteriorly by the psoas major and posteriorly by the erector spinae. After skin preparation and disinfection, a 20-G block needle (0.85 × 100 mm, Contiplex®; B. Braun, Tokyo, Japan) was inserted in-plane under ultrasound guidance from posterior to anterior through the quadratus lumborum muscle until the ventral fascia was punctured. Correct block needle tip placement was confirmed by instilling 1-2 mL of normal saline into the interfascial plane between the quadratus lumborum and psoas major and observing appropriate dispersion. After confirmation, 15 mL of ropivacaine (7.5 mg/mL, Anapeine®; AstraZeneca, Cambridge, UK) was injected.

Local infiltration analgesia

For both groups, LIA was administered by the surgeon at wound closure using a 20-G Contiplex needle (0.85 × 100 mm; B. Braun). LIA was performed according to an institutional standardized protocol throughout the study period. The injection targets and solution components were consistent, and no procedural changes to LIA were implemented during the study period. The solution was infiltrated into deep tissues, including the gluteus medius, tensor fasciae latae, intra-articular structures, joint capsule, fascia, and subcutaneous layers. The injection cocktail comprised 10 mL of ropivacaine (7.5 mg/mL), 10 mL of tranexamic acid (1,000 mg/10 mL), and 125 mg of methylprednisolone.

Intravenous patient-controlled analgesia (IV-PCA)

Postoperatively, all patients received IV-PCA. Before surgery, each patient was instructed on the proper use of the PCA device. The IV-PCA solution was prepared by mixing fentanyl citrate injection (0.5 mg/10 mL; Terumo Corporation, Tokyo, Japan) 20 mL with normal saline 80 mL to a total volume of 100 mL (final fentanyl concentration, 10 µg/mL). The PCA pump was programmed without a basal (continuous) infusion. Patients could self-administer 2 mL bolus doses on demand (20 µg fentanyl per bolus), with a 10-minute lockout interval and a maximum of five boluses per hour. Patients were instructed to use bolus doses as needed to maintain adequate pain control.

Additional perioperative medications

Postoperatively, all patients received scheduled oral acetaminophen 500 mg three times daily for seven days as part of a multimodal analgesic regimen. PONV was assessed during the first 48 hours postoperatively. Rescue antiemetic medication was administered when patients reported nausea within this period, and any use of antiemetic medication was recorded as a PONV event.

Clinical outcomes

As early mobilization is a key component of postoperative recovery after THA, pain severity during movement was assessed at 24 and 48 hours postoperatively using the NRS, a validated tool ranging from 0 (no pain) to 10 (worst imaginable pain). NRS scores were recorded during routine postoperative mobilization/rehabilitation assessments at 24 and 48 hours. Cumulative IV-PCA consumption was obtained from the PCA device records at 24 hours (0-24 h) and 48 hours (0-48 h). PONV within 48 hours was defined as any use of rescue antiemetics during the first 48 hours after surgery, as described above. Functional recovery was evaluated at discharge using the Japanese Orthopedic Association Hip-Disease Evaluation Questionnaire (JHEQ) [26].

Safety assessment

Postoperative lower-extremity motor function was assessed to detect potential neurologic adverse events. On postoperative day 1, the operating surgeon evaluated muscle strength using manual muscle testing (MMT), focusing on hip flexion and knee extension, and graded strength according to the Medical Research Council scale (0-5). Any motor deficits documented during this assessment were recorded as neurologic complications.

Patients were monitored for local anesthetic systemic toxicity (LAST) by an attending anesthesiologist during the perioperative period. Suspected LAST was defined as the occurrence of neurologic symptoms (e.g., tinnitus, perioral numbness, metallic taste, agitation, and seizures) and/or cardiovascular signs (e.g., arrhythmia, hypotension, and cardiac arrest) temporally related to local anesthetic administration. Events were identified from anesthesia and postoperative clinical records.

To balance analgesic efficacy with safety in the setting of concomitant ropivacaine-containing LIA, the QLB3 injectate volume was set at 15 mL. To minimize the risk of systemic toxicity, the total ropivacaine dose from QLB3 and LIA was calculated for each patient on a mg/kg basis and kept at ≤3.0 mg/kg. Because LIA contained 75 mg of ropivacaine (10 mL of 7.5 mg/mL), the QLB3 ropivacaine volume was adjusted in patients with lower body weight by reducing the injected volume in 1-2 mL increments to ensure that the combined dose did not exceed 3.0 mg/kg (minimum QLB3 volume: 6 mL).

Statistical analysis

All statistical analyses were performed using SPSS Statistics version 28.0 (IBM Corp., Armonk, NY). Continuous variables are presented as mean ± standard deviation (SD). Normality of continuous variables was assessed using the Shapiro-Wilk test. Between-group comparisons of continuous variables were performed using the Student’s t-test. Categorical variables are presented as numbers (%) and were compared using the chi-square test or Fisher’s exact test, as appropriate. For the primary outcome, NRS pain scores at 24 and 48 hours postoperatively were compared between groups at each time point. To account for repeated measures, a sensitivity analysis using a linear mixed-effects model was performed with fixed effects for group, time, and their interaction and a random intercept for each patient. Secondary outcomes, including cumulative IV-PCA consumption, incidence of PONV, and JHEQ scores at discharge, were analyzed using the same approach. Results are presented as mean differences or risk ratios with 95% confidence intervals (CIs). Statistical significance was set at p < 0.05 (two-tailed). No correction for multiple comparisons was applied, as analyses were exploratory. For transparency, adjusted p-values for secondary/exploratory outcomes were also calculated using the Holm-Bonferroni method (and Bonferroni correction) and are provided in the supplementary table. No formal a priori sample size calculation was performed because this was a retrospective exploratory study; the sample size was determined by the number of consecutive eligible patients during the study period.

## Results

**Figure 2:**
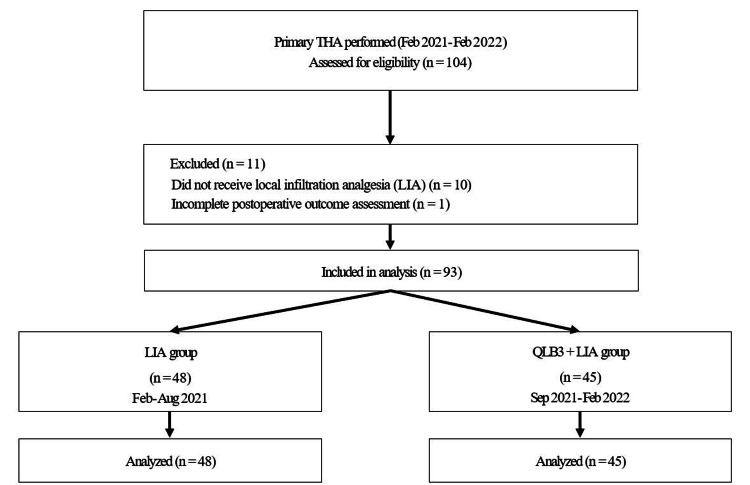
Flow diagram of patient selection and group allocation THA: Total hip arthroplasty; QLB3: Transmuscular quadratus lumborum block; LIA: Local infiltration analgesia.

Between February 2021 and February 2022, 104 patients underwent primary THA at our institution. Of these, patients who did not receive LIA (n = 10) and those with incomplete postoperative outcome assessments (n = 1) were excluded (Figure 2).

The remaining 93 patients constituted the analytic cohort. The mean age was 65.3 years (range: 39-88), with 15 men and 78 women. Average body mass index (BMI) was 24.5 kg/m² (range: 17.0-39.0 kg/m²) (Table 1). QLB3+LIA group (n = 45) and LIA group (n = 48) had comparable baseline characteristics, including age, BMI, operative time, and intraoperative blood loss (Table 1).

**Table 1:** Patient characteristics Data are expressed as mean ± standard deviation (range). QLB: Quadratus lumborum block; LIA: Local infiltration analgesia; BMI: Body mass index.

Variables	QLB3＋LIA	LIA	P-value
	(n = 45)	(n = 48)	
Sex (male:female)	6:39	9:39	0.48
Age (years)	66 ± 8.2 (30–82)	64.7 ± 11.6 (39–88)	0.61
Height (cm)	153.6 ± 6.7 (141.9–168.2)	155.6 ± 8.9 (135–173.4)	0.21
Weight (kg)	60.0 ± 11.8 (42–86.5)	58.1 ± 11.3 (40–96.6)	0.40
BMI (kg/m²)	25.3 ± 4.4 (18.9–36)	23.9 ± 4.1 (17–39)	0.23
Operation time (minutes)	152.7 ± 43.5 (80–330)	151.7 ± 30.6 (108–251)	0.91
Blood loss (ml)	329.7 ± 234.3 (50–1290)	317.7 ± 191.4 (50–850)	0.75

Postoperative NRS pain scores were significantly lower in the QLB3+LIA group than in the LIA group at both 24 and 48 hours (Table 2). At 24 hours, the mean NRS score was 2.4 ± 1.6 in the QLB3+LIA group and 4.2 ± 2.1 in the LIA group (p < 0.001). At 48 hours, the corresponding values were 2.4 ± 2.0 and 4.3 ± 2.1, respectively (p < 0.001). Sensitivity analyses using a linear mixed-effects model accounting for repeated measures at 24 and 48 hours yielded similar conclusions to the primary analyses.

**Table 2:** Overall comparison of outcomes Primary outcome: NRS pain intensity at 24 and 48 h. Secondary/exploratory outcomes: IV-PCA 24 h, IV-PCA 48 h, PONV, and JHEQ. Data are expressed as mean ± standard deviation. PONV effect estimate is the risk ratio (QLB3+LIA/LIA); other effect estimates are mean differences (QLB3+LIA - LIA). Differences are considered statistically significant at p < 0.05. QLB: Quadratus lumborum block; LIA: Local infiltration analgesia; NRS: Numerical rating scale; IV-PCA: Intravenous patient-controlled analgesia; PONV: Postoperative nausea and vomiting; JHEQ: Japanese Orthopaedic Association Hip-Disease Evaluation Questionnaire.

Outcome	QLB3＋LIA (n = 45)	LIA (n = 48)	Effect estimate	95% CI		P-value
				Lower	Upper	
24 h NRS	2.4 ± 1.6	4.2 ± 2.1	-1.78	-2.56	-1.01	<0.001
48 h NRS	2.4 ± 2.0	4.3 ± 2.1	-1.89	-2.73	-1.05	<0.001
24 h IV-PCA (ml)	9.5 ± 10.2	8.7 ± 10.9	0.83	-3.54	5.19	0.71
48 h IV-PCA (ml)	14.9 ± 15.1	18.2 ± 21.1	-3.28	-10.87	4.32	0.39
PONV (patients)	6/45 (13.3%)	16/48 (33.4%)	0.40	0.17	0.93	0.02
JHEQ (points)	40.4 ± 13.6	41.4 ± 15.8	-1.02	-7.11	5.07	0.74

Model-based fixed-effect estimates (group, time, and group × time interaction) with 95% CIs are presented in Table 3. Cumulative IV-PCA consumption at 24 hours did not differ between groups (9.5 ± 10.2 mL vs. 8.7 ± 10.9 mL; p = 0.71). Cumulative IV-PCA consumption at 48 hours was lower in the QLB3+LIA group than in the LIA group, but the difference was not statistically significant (14.9 ± 15.1 mL vs. 18.2 ± 21.1 mL; p = 0.39). PONV occurred less frequently in the QLB3+LIA group than in the LIA group (6/45 (13.3%) vs. 16/48 (33.3%); p = 0.02). JHEQ scores at discharge were similar between groups (40.4 ± 13.6 vs. 41.4 ± 15.8; p = 0.74). Adjusted p-values for secondary/exploratory outcomes are presented in the supplementary table.

**Table 3:** Linear mixed-effects model estimates for repeated postoperative outcomes Models included fixed effects for group, time, and group × time interaction, with a random intercept for patient. Reference categories: LIA for group and 24 h for time. Negative estimates indicate lower values in the comparison category. NRS estimates are in points, and IV-PCA estimates are in mL. The row "Model-derived between-group difference at 48 h" is a linear contrast of the group main effect plus the group × time interaction. IV-PCA: Intravenous patient-controlled analgesia; LIA: Local infiltration analgesia; NRS: Numeric rating scale; QLB: Quadratus lumborum block.

Outcome	Fixed effect/Contrast	Estimate	95% CI	P-value
NRS (points)	Group (QLB+LIA vs LIA, reference time = 24 h)	-1.785	-2.584 to -0.985	<0.001
	Time (48 h vs 24 h, reference group = LIA)	0.104	-0.489 to 0.698	0.731
	Group × time interaction	-0.104	-0.957 to 0.749	0.811
	Model-derived between-group difference at 48 h	-1.889	-2.688 to -1.089	<0.001
Cumulative IV-PCA (mL)	Group (QLB+LIA vs LIA, reference time = 24 h)	0.829	-5.282 to 6.941	0.790
	Time (48 h vs 24 h, reference group = LIA)	9.442	6.241 to 12.643	<0.001
	Group × time interaction	-4.108	-8.710 to 0.494	0.080
	Model-derived between-group difference at 48 h	-3.279	-9.390 to 2.833	0.293

## Discussion

In this retrospective single-center comparative study, the addition of ​​​​​an ultrasound-guided transmuscular QLB3 to LIA was associated with improved early postoperative analgesia after primary THA. Patients receiving QLB3 plus LIA reported significantly lower movement-evoked NRS pain scores at both 24 and 48 hours. Cumulative IV-PCA consumption within 48 hours was not significantly different between groups, although point estimates favored the QLB3 plus LIA group. Secondary outcomes, including PONV, should be interpreted cautiously, given multiple testing. Functional recovery at discharge, assessed using the JHEQ, was similar between groups, and we observed no block-related complications, including clinically apparent motor weakness or suspected LAST.

Beyond statistical significance, clinical relevance should be considered when interpreting postoperative pain outcomes. In our cohort, the between-group difference in NRS pain scores (approximately 1.8-1.9 points on a 0-10 scale at 24-48 h) appears comparable to commonly used minimal clinically important difference (MCID) benchmarks reported in arthroplasty pain research. In a systematic review of 570 THA/TKA analgesia trials, the median MCID for pain was 15 mm at rest and 18 mm during movement on a 0-100 mm visual analog scale (VAS), and the median MCID for 0-24 h opioid consumption was 10 mg intravenous morphine equivalents; importantly, 46% of trials with statistically significant primary outcomes did not reach the predetermined MCID [27]. In a postoperative arthroplasty cohort, the MCID for improving VAS pain after THA during hospitalization was estimated at −18.6 mm [28]. Although MCID thresholds vary by scale, timing, and population, these data suggest that the magnitude of pain reduction observed with QLB3 plus LIA in our study may be clinically meaningful, not merely statistically significant.

The current evidence for QLB in THA is heterogeneous, which may partially explain why its role remains debated. He et al. reported that a transmuscular QLB3 performed before spinal anesthesia significantly reduced postoperative pain scores at rest and during mobilization through 48 hours, decreased morphine consumption, improved 10‑m walking speed, and reduced nausea and vomiting compared with placebo [19]. In contrast, Brixel et al. found that adding a posterior QLB to a multimodal analgesia regimen under general anesthesia did not reduce 24-hour morphine consumption or pain scores compared with saline [29]. A randomized trial comparing anterior QLB with femoral nerve block demonstrated similar analgesia at rest and no difference in 24-hour morphine consumption, concluding that whether anterior QLB is inferior or noninferior to femoral nerve block was inconclusive [30]. Differences in QLB approach (transmuscular versus posterior versus anterior), local anesthetic dose and spread, background multimodal regimens, and anesthetic techniques likely contribute to these discordant findings.

Many published QLB protocols use larger injectate volumes (e.g., 20-30 mL) [15,29], whereas we used a lower volume because QLB3 was combined with ropivacaine-containing LIA and we aimed to balance analgesic efficacy with safety. We acknowledge that a lower volume may influence the extent of spread and dermatomal coverage. Nevertheless, meaningful analgesic benefit was observed in our cohort. Future prospective studies should evaluate the volume-effect relationship and determine the optimal dosing strategy when QLB is used in combination with LIA.

Systematic reviews and meta-analyses provide a similarly tempered interpretation. Hussain et al. analyzed 18 randomized trials and interpreted pain outcomes against a population-specific MCID of 1.86 cm on a 10‑cm VAS [31]. While QLB improved 24-hour area under the curve rest pain scores, the effect did not reach the prespecified MCID, and reductions in analgesic consumption were also below MCID thresholds. Accordingly, the authors did not support routine QLB use as part of multimodal analgesic regimens for THA, although opioid-related side effects were reduced [31]. Gazendam et al. included seven randomized trials and reported no differences in pain scores or postoperative opioid consumption between QLB and control interventions, with rare and similar adverse events between groups [32]. Behera et al. also reported a systematic review and meta-analysis evaluating single-shot QLB for postoperative analgesia in adults undergoing THA [33]. In the context of contemporary procedure-specific guidance, the PROSPECT guideline for THA emphasizes basic multimodal analgesia (paracetamol and non-steroidal anti-inflammatory drugs/cyclooxygenase-2 inhibitors), recommends intraoperative intravenous dexamethasone 8-10 mg, and recommends regional options such as fascia iliaca block or LIA, while noting limited or inconsistent evidence for other approaches [34]. Taken together, our real-world findings suggest that QLB3 may provide an additive benefit when combined with LIA in selected THA patients; however, given the conflicting randomized controlled trial and meta-analytic evidence, further prospective trials powered for clinically meaningful effect sizes (MCID-based) and reporting functional recovery and safety outcomes are warranted to define which patients derive the greatest benefit.

Safety is a key consideration when selecting regional techniques after THA, particularly in the setting of early mobilization protocols. In our practice, lower-extremity motor function was assessed by the operating surgeon on postoperative day 1 using MMT, and patients were monitored by anesthesiologists for symptoms and signs suggestive of LAST; no clinically apparent motor deficits or suspected LAST events were documented. Nevertheless, because our safety assessments were based on routine clinical evaluation rather than serial quantitative strength testing and standardized neurologic examinations, subtle or transient effects may have been missed.

This study has several limitations. First, its retrospective before-after design introduces potential selection bias and residual confounding. Because group allocation was determined by time period, our findings may also be affected by temporal confounding (secular trends), such as unmeasured changes in perioperative pathways, staffing, or institutional practice over time, even if measured baseline characteristics were similar between groups. Second, the sample size was relatively small, and further evaluation in larger, multicenter cohorts is required. Third, because multiple outcomes were assessed and no adjustment for multiple comparisons was applied, there is a risk of inflated type I error; therefore, findings for secondary/exploratory outcomes should be interpreted cautiously. Fourth, surgical approaches were not standardized; for example, the direct lateral (Hardinge) approach involves splitting the gluteus medius muscle, whereas other approaches are intermuscular, resulting in variable muscle trauma that may affect postoperative pain. Fifth, we assessed patient-reported hip function using the JHEQ at hospital discharge; however, the JHEQ was originally developed to capture hip-related symptoms and function over the preceding three months. Therefore, JHEQ scores obtained at discharge may not precisely reflect very early postoperative recovery and should be interpreted with caution [26]. Finally, pain intensity was assessed only during movement in routine postoperative mobilization/rehabilitation, and rest pain scores were not systematically recorded in the medical records. Therefore, our findings may not be directly comparable with prior studies that reported both rest and movement pain outcomes.

## Conclusions

In this single-center retrospective before-after cohort, QLB3 combined with LIA was associated with lower movement-evoked pain scores at 24 and 48 hours after primary THA, without documented block-related complications. IV-PCA consumption within 48 hours was not significantly different between groups, and secondary outcomes, including PONV, should be interpreted cautiously. Given the nonrandomized before-after design and the possibility of temporal confounding, these findings should be considered hypothesis-generating. Prospective randomized studies with standardized perioperative protocols and clinically meaningful endpoints are needed to confirm efficacy, define optimal dosing when QLB is combined with LIA, and further evaluate safety.

## Appendices

**Table 4:** Adjusted p-values for secondary/exploratory outcomes After Holm or Bonferroni correction, none of the secondary/exploratory outcomes remained statistically significant at an alpha level of 0.05. The PONV sensitivity analysis (Fisher’s exact test) is presented as an unadjusted sensitivity analysis for the same PONV endpoint and was not included as a separate outcome in the Holm-Bonferroni or Bonferroni multiplicity adjustment. IV-PCA: Intravenous patient-controlled analgesia; PONV: Postoperative nausea and vomiting; JHEQ: Japanese Orthopaedic Association Hip-Disease Evaluation Questionnaire.

Secondary Outcome	Unadjusted P-Value	Holm Adjusted P-Value	Holm Significant at 0.05	Bonferroni Adjusted P-Value	Bonferroni Significant at 0.05
IV-PCA 24 h (mL)	0.7067	1.0000	False	1.0000	False
IV-PCA 48 h (mL)	0.3933	1.0000	False	1.0000	False
PONV (chi-square)	0.0233	0.0933	False	0.0933	False
JHEQ (points)	0.7401	1.0000	False	1.0000	False
PONV sensitivity analysis					
Fisher's exact P-value	0.0289				
